# *IGF2, LEPR*, *POMC*, *PPARG*, and *PPARGC1* gene variants are associated with obesity-related risk phenotypes in Brazilian children and adolescents

**DOI:** 10.1590/1414-431X20154155

**Published:** 2015-04-28

**Authors:** E.M. Queiroz, A.P.C. Cândido, I.M. Castro, A.Q.A. Bastos, G.L.L. Machado-Coelho, R.N. Freitas

**Affiliations:** 1 Departamento de Nutrição Clínica e Social, Núcleo de Pesquisas em Ciências Biológicas, Universidade Federal de Ouro Preto, Ouro Preto, MG, Brasil; 2 Departamento de Nutrição, Universidade Federal de Juiz de Fora, Juiz de Fora, MG, Brasil; 3 Departamento de Farmácia, Núcleo de Pesquisas em Ciências Biológicas, Universidade Federal de Ouro Preto, Ouro Preto, MG, Brasil; 4 Escola de Medicina, Núcleo de Pesquisas em Ciências Biológicas, Universidade Federal de Ouro Preto, Ouro Preto, MG, Brasil

**Keywords:** Association study, Obesity, Genetic polymorphisms, Brazilian population

## Abstract

Association studies of genetic variants and obesity and/or obesity-related risk factors have yielded contradictory results. The aim of the present study was to determine the possible association of five single-nucleotide polymorphisms (SNPs) located in the *IGF2*, *LEPR*, *POMC*, *PPARG*, and *PPARGC1*genes with obesity or obesity-related risk phenotypes. This case-control study assessed overweight (n=192) and normal-weight (n=211) children and adolescents. The SNPs were analyzed using minisequencing assays, and variables and genotype distributions between the groups were compared using one-way analysis of variance and Pearson's chi-square or Fisher's exact tests. Logistic regression analysis adjusted for age and gender was used to calculate the odds ratios (ORs) for selected phenotype risks in each group. No difference in SNP distribution was observed between groups. In children, *POMC rs28932472*(*C*) was associated with lower diastolic blood pressure (P=0.001), higher low-density lipoprotein (LDL) cholesterol (P=0.014), and higher risk in overweight children of altered total cholesterol (OR=7.35, P=0.006). In adolescents, *IGF2 rs680*(*A*) was associated with higher glucose (P=0.012) and higher risk in overweight adolescents for altered insulin (OR=10.08, P=0.005) and homeostasis model of insulin resistance (HOMA-IR) (OR=6.34, P=0.010). *PPARG *rs1801282(*G*) conferred a higher risk of altered insulin (OR=12.31, P=0.003), and HOMA-IR (OR=7.47, P=0.005) in overweight adolescents. *PARGC1 rs8192678*(*A*) was associated with higher triacylglycerols (P=0.005), and *LEPR rs1137101*(*A*) was marginally associated with higher LDL cholesterol (P=0.017). *LEPR rs1137101*(*A*) conferred higher risk for altered insulin, and HOMA-IR in overweight adolescents. The associations observed in this population suggested increased risk for cardiovascular diseases and/or type 2 diabetes later in life for individuals carrying these alleles.

## Introduction

Individuals who are overweight or obese are at significantly greater risk for death (1). Specifically, the two conditions are risk factors for type 2 diabetes, cardiovascular diseases (2,3), many forms of cancer (4), pulmonary disease, hypertension (2,3), dyslipidemia, and osteoarticular and psychiatric diseases (3).

Obesity is due to an imbalance between food intake and energy expenditure that is determined by environmental and genetic factors. Eighteen genes associated with obesity have already been identified by genome-wide association studies (5-7). Other genes are unequivocally associated with factors related to obesity (8-14); however, the associations of some of these genes with obesity have been inconclusive, and few studies have investigated subjects with an onset of obesity at an early age. Moreover, there is a lack of data on populations from Southern Hemisphere countries, especially for children and adolescents.

To determine the association of genetic variants and obesity and/or obesity-related risk factors, we analyzed the genotype and allele distributions of five single-nucleotide polymorphisms (SNPs) located in the genes for insulin-like growth factor 2 (*IGF2*), leptin receptor (*LEPR*), proopiomelanocortin (*POMC*), peroxisome proliferator-activated receptor gamma (*PPARG*), and peroxisome proliferator-activated receptor gamma coactivator 1 (*PPARGC1*) in samples of overweight and normal-weight children and adolescents in a mixed population from southeastern Brazil. We also analyzed the associations of the SNPs with obesity-related risk phenotypes, which are metabolic syndrome components.

## Material and Methods

### Study design

A case-control study was conducted using a sample obtained from a cross-sectional population-based study, carried out with children and adolescents aged 7 to 14 years from all schools (14 public and 2 private schools) in the urban zone of Ouro Preto city, State of Minas Gerais, southeastern Brazil between 2008 and 2012 (15). The case group was composed of overweight individuals. The control group, paired by gender and age, was selected from a list of eutrophic individuals according to the order entered in the cross-sectional study. The selection of volunteers was made by simple random selection stratified by the proportion of students grouped according to age, gender, and school. Students with special needs were not included. The sample size was calculated considering the prevalence of overweight status (8%) reported for the population in the age group of the study, an estimated accuracy of 3%, estimated loss of 20%, and a significance level of 95%. Demographic, biochemical, clinical, and anthropometric data were collected. The tetrapolar bioelectrical impedance method was used to assess body fat percent as calculated by Deurenberg et al. (16). Subjects aged 7 to 14 years were classified according to gender-specific 75th percentile of body fat percentage.

Individuals were categorized according to cutoff values proposed for children and adolescents for some obesity-related risk phenotypes: glucose and waist circumference as proposed by the International Diabetes Force consensus (17), and body mass index (BMI), total cholesterol, low-density lipoprotein (LDL) cholesterol, high-density lipoprotein (HDL) cholesterol, triacylglycerides, blood pressure, and insulin as determined by the Brazilian Society of Cardiology (18). Insulin resistance was estimated by the homeostasis model of insulin resistance (HOMA-IR) (19) and was considered high when HOMA-IR >3.16 (20). Because all participants were underage, their legal guardians signed a consent form, and the project was approved by the Research Ethics Committee of the Universidade Federal de Ouro Preto (No. 0017.238.000-05).

### Genotyping assay

Genomic DNA was obtained from a blood sample according to Miller et al. (21). The selection of polymorphisms assessed in this study was in accordance with the following criteria: 1) positive association with obesity in at least five previous studies and ethnic groups related to the formation of the population of Minas Gerais, 2) no rare allele, and 3) involves exchanges by guanine or cytosine. Gene fragments were co-amplified (5 µL) with 100 ng DNA, 0.4 µM of each primer (c) and 1× Qiagen Multiplex PCR Master Mix commercial kit (Qiagen, The Netherlands). The polymerase chain reaction (PCR) conditions were 15 min at 95°C, 39 cycles of 30 s at 94°C, 90 s at 57°C, 60 s at 72°C, and 10 min at 72°C. After amplification, 2 µL of the PCR product was digested by enzymatic solution containing 2 U/µL of *Escherichia coli* exonuclease I (Fermentas Life Sciences, USA), 0.2 U/µL shrimp alkaline phosphatase (Fermentas Life Sciences), and 1× shrimp alkaline phosphatase buffer and then incubated at 37°C for 30 min followed by 15 min at 80°C. The SNP allele identification (5 µL) was 1 µL digested PCR product; 0.01-0.6 µM of each primer (Supplementary Table S1); 3.5 mM MgCL_2_, 1× Thermo Sequenase DNA polymerase buffer; 0.5 µM fluorescein-labeled 2,3-dideoxycytidine-5′ triphosphate (ddCTP) (PerkinElmer Life and Analytical Sciences, USA); 0.5 µM each unlabeled deoxyguanosine triphosphate (dGTP), deoxythymidine triphosphate (dTTP), and deoxyadenosine triphosphate (dATP); and 1 U Thermo Sequenase DNA Polymerase (GE Healthcare, UK). The reaction conditions were 5 min at 80°C, 30 cycles of 30 s at 95°C, 30 s at 55°C, 20 s at 72°C, and 5 min at 72°C. The monochrome electrophoresis was conducted in a MegaBace 1000 sequencer (GE Healthcare). Data were analyzed using the Fragment Profiler software (GE Healthcare).

### Statistical analysis

Insulin values were log_10_ transformed to approximate normal distribution. To test for differences between normal-weight and overweight subjects and between genotype groups, we used one-way analysis of variance (ANOVA) for continuous variables and Pearson's chi-square or Fisher's exact tests for categorical variables. Genotype frequencies were tested for Hardy-Weinberg equilibrium. Logistic regression analysis adjusted by age and gender was used to calculate the odds ratios (ORs) for selected phenotype risks associated with obesity and metabolic syndrome in each normal-weight and overweight group. Statistical analyses were performed using the SPSS version 18.0 software (SPSS Inc., USA). Significance level was set at P≤0.05, except for multiple comparisons, in which P values were adjusted using Bonferroni's correction (P≤0.01).

## Results

As expected, mean weight (P<0.001), BMI (P<0.001), waist circumference (P<0.001), systolic blood pressure (P=0.012), diastolic blood pressure (P=0.013), triacylglycerides (P=0.030), insulin (P=0.008), and HOMA-IR (P=0.018) were higher in overweight individuals than in normal-weight individuals. There were no differences in other continuous variables and gender between the overweight and normal-weight groups (Table 1).

**Table:**
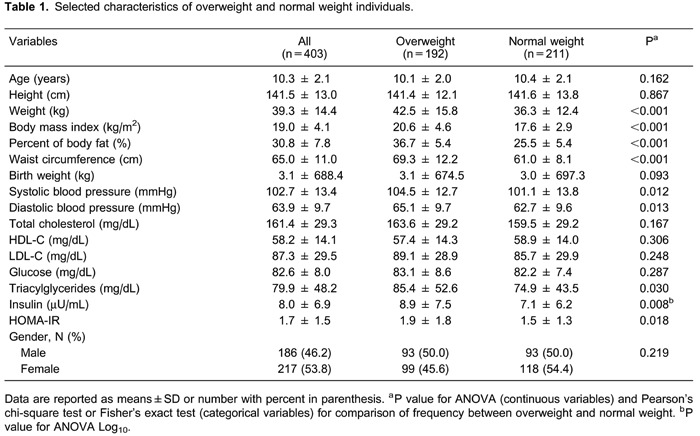

Table 2 shows the genotype and allele distributions of the five SNPs in all individuals and in the overweight and normal-weight groups. There were no differences in genotype and allele distributions. The genotype distributions of all SNPs were in Hardy-Weinberg equilibrium.

**Table:**
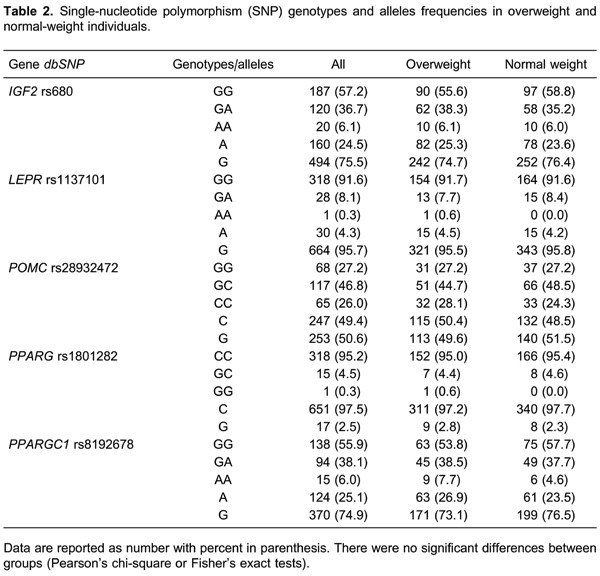

Table 3 summarizes the comparison of the anthropometric, clinical, and biochemical variables between the groups for the SNPs that showed significant association with at least one obesity-related risk phenotype for children or adolescents. In children, subjects with the *POMC*rs28932472 allele *C* presented lower diastolic blood pressure (P=0.001) and higher LDL cholesterol (P=0.014) than *G* homozygous alleles. The *IGF2* rs680 allele *A* was associated with higher glucose (P=0.012) concentrations than measured in *G*homozygous alleles. The *PPARGC1* rs8192678 allele *A*presented higher triacylglycerol (P=0.005) concentrations than *G*homozygous alleles. Subjects with the *LEPR* rs1137101 allele *A* presented higher LDL cholesterol concentrations than *G* homozygous alleles that were only marginally significant (P=0.017). In adolescents, only the *POMC* rs28932472 allele *C* was associated with lower glucose (P=0.009) concentrations than *G* homozygous subjects. No association was found for *PPARG* rs1801282 or the other variables tested (BMI, body fat percentage, waist circumference, birth weight, systolic blood pressure, total cholesterol, LDL/HDL cholesterol, insulin, and HOMA-IR).

**Table:**
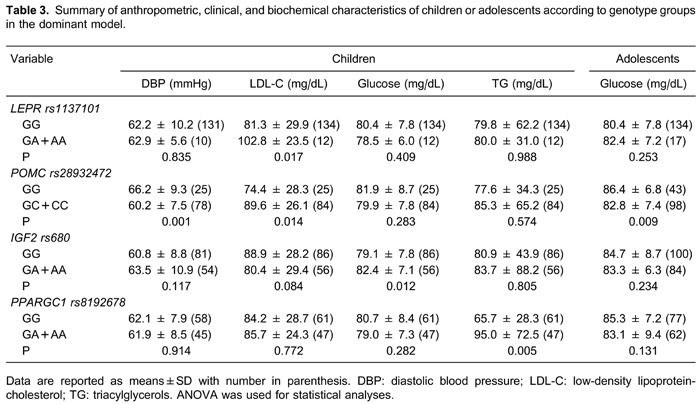

Table 4 shows the results of the logistic regression for the obesity-related risk phenotype for *IGF2* rs680, *LEPR* rs1137101, *PPARG* rs1801282, and *POMC* rs28932472 in normal and overweight children or adolescents, with the common homozygous allele as reference (dominant model). With respect to lipid profile, overweight children carrying the *C* allele for *POMC* rs28932472 polymorphism had higher odds for higher total cholesterol (OR=7.35, 95% confidence interval [CI]=1.77-30.49, P=0.006). Additionally, overweight adolescents carrying the *A* allele for *IGF2* rs680 or the *A* allele for *LEPR* rs1137101 or the *G* allele for *PPARG* rs1801282 polymorphism had higher odds for higher insulin (OR=10.08, 95%CI=1.99-51.04, P=0.005; OR=10.31, 95%CI=2.07-51.27, P=0.004; and OR=12.31, 95%CI=2.31-65.50, P=0.003, respectively) and higher odds for higher HOMA-IR (OR=6.34, 95%CI=1.56-25.81, P=0.010; OR=6.51, 95%CI=1.64-25.86, P=0.008; and OR=7.47, 95%CI=1.82-30.72, P=0.005, respectively).No association was found for *PPARGC1* rs8192678.

**Table:**
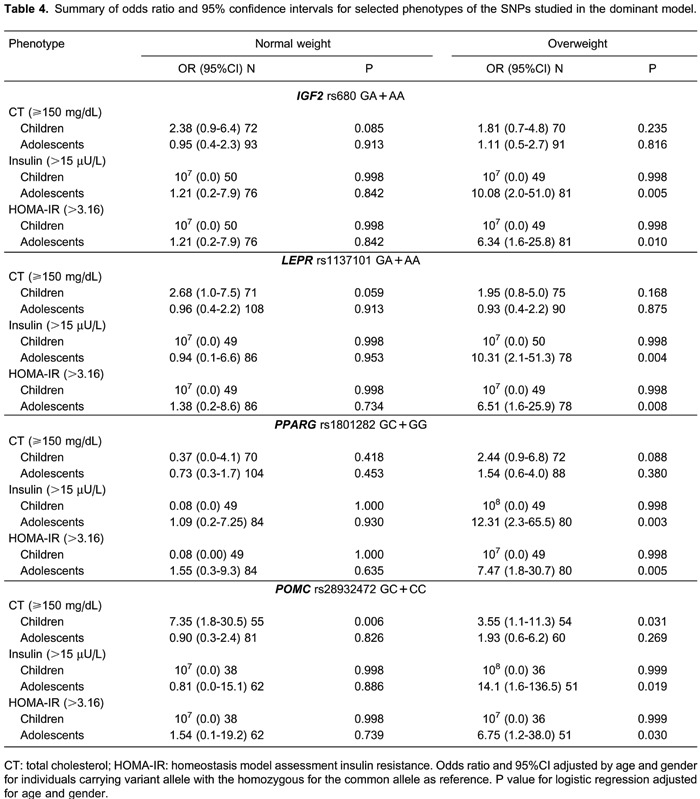

## Discussion

In children and adolescents, BMI is the traditional method used to characterize nutritional status (22). However, it does not provide information on the proportions of fat and lean masses, so other methods have been used to infer the body composition of children and adolescents, such as skinfold thicknesses, body circumferences, bioelectrical impedance analysis, and dual-energy X-ray absorptiometry (23). In fact, there are few nutrigenetics studies of Southern Hemisphere populations, especially in children and adolescents (24,25). Because genetic ancestral background appears to contribute to the variation in adiposity at the population level (26), and gene-environment interactions account for risk phenotypes, the results of nutrigenetics studies can only be applied or extrapolated in well characterized populations. Thus, our study contributes to studies on mixed populations.

Although we did not find any association between the five SNPs and adiposity, other risk phenotypes related to obesity were associated with *LEPR*rs1137101, *POMC* rs28932472, *IGF2* rs680, *PPARG* rs1801282, and *PPARGC1* rs8192678.

We found that *LEPR* rs1137101 was associated with higher LDL cholesterol values in adolescents. Studies with children and adolescents did not reveal an association between this SNP and HDL or LDL cholesterol values, waist circumference, body fat percentage, insulin, triacylglycerides, glucose, total cholesterol, HOMA-IR, or blood pressure (27,28). Conflicting results have been reported for BMI (27,28), and higher daily energy intake was observed in Brazilian children at 4 years of age (29). Even though some studies showed an association between *POMC* rs28932472 and early age of obesity onset in children and adolescents (30,31), there is little information about the phenotypes associated with this SNP. We observed associations between *POMC*rs28932472 and lower diastolic blood pressure and higher LDL cholesterol values in adolescents. Additionally, we observed an association of this SNP with lower glucose in children. In a study with Italian children and adolescents, the LDL cholesterol values for heterozygous individuals were similar to those found in our study (32). We also found that *IGF2*rs680 was associated with higher glucose in adolescents. In Brazilian adults, this SNP was associated with BMI and birth weight (33). We also observed an association between *PPARGC1*rs8192678 and higher triacylglycerol concentration in adolescents, which is similar to the results of an adult study (34). Although we did not observe an association of *PPARG* rs1801282 with clinical, biochemical, or anthropometric characteristics in our study, the association of this SNP with higher glucose was reported in Brazilian children at 4 years of age (29).

The association of these five SNPs with obesity-related risk phenotypes has not been routinely investigated in children and adolescents. We found that the *LEPR* rs1137101, *IGF2* rs680, and *PPARG* rs1801282 SNPs were associated with higher ORs for insulin and HOMA-IR, and *POMC* rs28932472 SNP was associated with a higher OR for total cholesterol. The relationship between *PPARG*rs1801282 with insulin and HOMA-IR is known because this SNP has been associated with type 2 diabetes (35,36). On the other hand, there is no information about an association of *IGF2* rs680 or *LEPR*rs1137101 with type 2 diabetes. Although the association of *POMC*rs28932472 with a higher OR for total cholesterol has not been previously reported, one study reported a higher prevalence of this SNP in obese individuals that characteristically tended to exhibit higher values of total cholesterol (31).

Genetics studies of obesity are often performed in Caucasian populations; little is known about the frequency of obesity-related polymorphisms in the admixture population. Thus, the present study provides new information about the frequency of these polymorphisms and risk in an admixture cohort such as the Brazilian population.

The study has some limitations. First, because the case and control groups were assessed in a cross-sectional study, the effects of the SNPs on the risk phenotypes over time are unknown. We also did not consider environmental factors such as diet or physical activity that might change the effect of the polymorphisms on the phenotypes. Lastly, we cannot rule out the possibility that the identified associations were due to chance, even though the analyses used to examine the relationship between candidate genotypes and risk phenotypes were based on *a priori* hypotheses. Nevertheless, this is an original study of an understudied population, and our results will help clarify the genetics of risk phenotypes associated with obesity in children and adolescents.

In conclusion, our results revealed associations between SNPs in candidate genes and obesity-related phenotypes in Brazilian children and adolescents, which could suggest increased risk for cardiovascular diseases or type 2 diabetes later in life for individuals carrying these alleles.

## Supplementary material

Click here to view [pdf]
